# The Global Health Policies of the EU and its Member States: A Common Vision?

**DOI:** 10.15171/ijhpm.2017.112

**Published:** 2017-10-02

**Authors:** Lies Steurs, Remco Van de Pas, Sarah Delputte, Jan Orbie

**Affiliations:** ^1^Centre for EU Studies, Ghent University, Gent, Belgium.; ^2^Institute of Tropical Medicine, Antwerp, Belgium.; ^3^Centre for Global Health Research, Maastricht University, Maastricht, The Netherlands.; ^4^Clingendael, Netherlands Institute of International Relations, The Hague, The Netherlands.

**Keywords:** European Union (EU), Global Health, Framing, Development Cooperation, Foreign Policy

## Abstract

**Background:** This article assesses the global health policies of the European Union (EU) and those of its individual member states. So far EU and public health scholars have paid little heed to this, despite the large budgets involved in this area. While the European Commission has attempted to define the ‘EU role in Global Health’ in 2010, member states are active in the domain of global health as well. Therefore, this article raises the question to what extent a common ‘EU’ vision on global health exists.

**Methods:** This is examined through a comparative framing analysis of the global health policy documents of the European Commission and five EU member states (France, Germany, the United Kingdom, Belgium, and Denmark). The analysis is informed by a two-layered typology, distinguishing global health from international health and four ‘global health frames,’ namely social justice, security, investment and charity.

**Results:** The findings show that the concept of ‘global health’ has not gained ground the same way within European policy documents. Consequently, there are also differences in how health is being framed. While the European Commission, Belgium, and Denmark clearly support a social justice frame, the global health strategies of the United Kingdom, Germany, and France put an additional focus on the security and investment frames.

**Conclusion:** There are different understandings of global/international health as well as different framings within relevant documents of the EU and its member states. Therefore, the existence of an ‘EU’ vision on global health is questionable. Further research is needed on how this impacts on policy implementation.

## Background

During the past 20 years health has increasingly gained importance on the global policy agenda. There has been an unprecedented growth in funding for health, several new partnerships and initiatives were launched^ [1] ^, philanthropic foundations such as the Bill and Melinda Gates Foundation became key players, and health emerged on the agenda of high-level fora such as the United Nations (UN) and the G8. This ‘global health revolution’ has also been accompanied by “a re-conceptualization of health as more than a technical, humanitarian concern and as relevant to the vital interests of states in security and economic well-being.”^1^ A milestone has been The Oslo Ministerial Declaration, advanced by the ministers of Foreign Affairs of Brazil, France, Indonesia, Norway, Senegal, South Africa, and Thailand in 2006, that declares that ‘‘health as a foreign policy issue needs a stronger strategic focus on the international agenda.”^2^

The European Union (EU) has been trying to find its place in the growing global health arena, in addition to the efforts of its member states.^3^ Health has always been an important issue in the development policy of the EU. In 2002 already, the European Commission’s communication on *Health and Poverty Reduction in Developing Countries* established for the first time “a single Community policy framework to guide future support for health, AIDS, population and poverty within the context of overall EC assistance to developing countries.”^4^ While recognizing the “differing histories and experiences in framing development policy”^4^ of member states, the increasing convergence of general development objectives was mentioned as an opportunity to improve coordination of EU Member states’ development policies and approaches in the health sector. Furthermore, the European Consensus on Development (2005) stressed the importance of the Millennium Development Goals (MDGs), with a specific focus on the health-related MDGs. Also in the EU’s health policy more attention has been given to global health issues. In 2007 the importance of a European contribution to the global health debate was recognized in a white paper stating that “in our globalized world, it is hard to separate national or EU-wide actions from the global sphere, as global health issues have an impact on internal community health policy and vice versa.”^5^

Recognizing that global health is influenced by several policy domains, the Directorate-General (DG) Health, DG Development and DG Research initiated a consultation process with several stakeholders in 2009, which resulted in the launch of a Commission communication on *the EU Role in Global Health* in 2010.^6^ This document stated that “the EU should apply the common values and principles of solidarity towards equitable and universal coverage of quality health services in all external and internal policies and actions.”^6^ By focusing on universal coverage of basic quality care, health systems strengthening and policy coherence, it proposed a clear vision on global health. This message was confirmed by subsequent Council conclusions.^7^

These attempts of the EU to claim a role in global health can be seen as part of ongoing efforts to ‘europeanize’ development policy. The European Commission is not only an international donor in its own right, it also aims to play a ‘federalizing’ role in coordinating and harmonizing the aid policies of its Member states. Since the 2000s, the European Commission has increasingly stressed this latter role, fostering European aims, European approaches and European actions in development policy.^8,9^

However, despite EU attempts to coordinate global health policies, member states want to keep a grip on this domain as well. The Council Conclusions on the EU role in Global Health make it very clear that the stronger EU voice on global health should be endeavored “without prejudice to the respective competencies.”^7^ As development policy is a shared competence, EU donors have their own policies regarding (health) development policy. Furthermore, the conceptual shift from ‘international health’ to ‘global health’ (infra) has not manifested itself in the same way along all EU Member states. Like the European Commission, some member states have released their own global health strategies (the United Kingdom in 2008 and 2011, Germany in 2013 and France in 2017). While the strategies are also the result of an interdepartmental cooperation, they might not necessarily echo the central objectives of the 2010 Commission communication. As member states remain important actors in development policy in general, and in global health more specifically, the question remains to what extent a common ‘EU’ vision on global health exists. This is precisely the research question of this article.

Gaining a better understanding of global health frames within the EU is important for several reasons. First, we concur with a social constructivist ontology that frames are not merely rhetorical acts: the framing of issues like global health has important power implications by determining policies and actions. A frame can be defined as “an organizing principle that transforms fragmentary or incidental information into a structured and meaningful problem, in which a solution is implicitly or explicitly included.”^10^ Framing analysis has been used extensively in social sciences.^11,12^ More recently, it has also been used in EU^10,13^ and global health studies.^14-18^ Despite methodological and theoretical differences, these studies share the idea that the same issue can be framed differently by different actors, and that the way a certain topic is ‘framed’ also affects the proposed solutions to deal with this topic. Therefore, it is important to better understand the frame(s) being used, even if it is beyond the scope of this study to examine the implementation of global health policies. It is all the more relevant to study the European Commission and its member states, as these are significant donors for global health funding with substantial budgets. Theoretically, we consider frames as a coherent set of ideas at the level of ‘policies’ (ie, defining policy problems), which is more abstract than ‘policy proposals’ (ie, responding to specific policy problems) but more specific than ‘values and ideologies’ (ie, overarching paradigms).^19^ As explained in the methodology section, the ‘policy frames’ are identified based on the European Commission and member states’ major strategic documents on international health and development.

Second, this research is relevant from an EU policy perspective, as it allows us to assess the European Commission’s objective to foster “a stronger EU vision, voice and action”^6^ in global health. Importantly, however, this does not mean that we aim to develop a normative ‘ideal’ EU approach against which the approaches of European donors must be balanced. From a normative perspective we do not claim that the existence of different frames would in itself be problematic and that a standardized definition would be necessary. While a diversity of frames would be problematic from an European Commission policy perspective, it may indeed be desirable from a normative democracy standpoint that stresses the importance of conflicting political values.

Third, this research fills a gap in the literature, as the relationship between global health policies of the EU and its member states has so far received little attention among EU and public health scholars. Studies on the EU’s role in global health are mostly confined to the European Commission’s policy^3,20,21^ and the EU’s representation in the World Health Organization (WHO).^22,23^ A recent edited volume provides the main policy approaches, case studies and a critical assessment on the EU role in Global Health.^24^ The policies of EU member states on global health have been largely neglected (^25,26^ are noteworthy exceptions).

By mapping and comparing the existing approaches of EU donors, we want to question whether a unified vision exists. On top of that, we will reflect on some possible implications of stressing one or another framing. Specifically, as will be discussed in the next section, we will examine relevant policy documents of the European Commission and five member states based on a two-layered typology of global/international health frames. Subsequently, the findings of the comparative analysis will be discussed. Lastly, the conclusions will summarize the main findings and discuss some suggestions for further research.

## Methods

Methodologically, in order to examine to what extent a common EU vision on global health exists from a framing perspective, we elaborated a typology of global health frames and applied it to relevant policy documents of the European Commission and five member states. The two-layered typology (see next section) was developed through an abductive research process.^27,28^ In line with our social constructivist approach, we do not assume that frames are pre-defined and exist independently from social reality. While the literature provided us with relevant frames, our framework has been adapted throughout the empirical analysis of the policy documents. Indeed, abduction reasons at an intermediate level between deduction (where a fixed framework is imposed) and induction (where findings are built on empirics).^27^ It involves a continuous interaction between theoretical and empirical research, whereby literature review, data generation, data analysis and research design mutually influence each other in a cyclical research process. As such abductive modes of inquiries fit nicely with constructivist approaches.

Applied to our research, a number of frames that are suggested in the literature were dropped because they proved less relevant throughout the empirical research. For instance, the ‘development frame’ was considered too general for this purpose of this research, as all frames concern the global health-development nexus in some way. The broader question is not so much whether global health is framed as relevant for development, but how global health and development are seen to be interrelated. Inversely, document analysis revealed that we should go beyond the distinction between ‘international’ and ‘global’ health that is often made in the literature, and delve deeper into the different ways in which both international and global health can be framed. Hence the two-layered typology which also constitutes a conceptual contribution to the literature (see below). Besides the European Commission, five member states were selected for our comparative analysis, namely: France, Germany, the United Kingdom, Belgium, and Denmark. This selection is based on several reasons. First, it includes the largest EU donors in terms of budgets for health in development. Second, it includes big and small member states, as well as Northern and Southern member states, thereby potentially covering a broad range of European approaches towards development.^29,30^ Third, these five donors each have issued a clear and explicit health strategy. Fourth, limiting the number of donors allows for an in-depth and comparative analysis, which is particularly important, as this has not yet been done in existing literature.

For each donor, relevant policy documents were identified. We systematically scanned sector strategies of the development/international cooperation department and/or ‘whole-of-government strategies’ of each donor, which allowed us to detect those strategy documents that explicitly address global/international health^ [2] ^. Table 1 provides an overview of the relevant documents.

**Table 1:** Overview of Analyzed Policy Documents

**European Commission**	**Communication on ‘the EU Role in Global Health’ (2010)**
United Kingdom	Health Is Global: A UK Government Strategy 2008-2013 UK Government. Health Is Global: An Outcomes Framework for Global Health 2011-2015 DFID Health Position Paper - Delivering Health Results (2013)
Germany	Sector Strategy: German Development Policy in the Health Sector (2009) Shaping Global Health - Taking Joint Action - Embracing Responsibility: The Federal Government's Strategy Paper (2013)
France	Strategy for International Health Cooperation (2012) The French Strategy in Global Health (2017)
Belgium	The Right to Health and Healthcare (2008)
Denmark	Health and Development, a Guidance Note to Danish Development Assistance to Health (2009) Strategy for Denmark’s Support to the International Fight against HIV/AIDS (2005) The Promotion of Sexual and Reproductive Health and Rights, Strategy for Denmark’s Support (2006)

Abbreviations: EU, European Union; DFID, Department for International Development.

In line with the abductive research process, the documents were analyzed first through a close reading and second by using NVivo software. First, and in line with major debates in the literature, it was analyzed whether the document could be considered to be an ‘international health’ or ‘global health’ document. This involved an analysis of the substantive content of the document and an examination of the institutional ownership. Second, we aimed to identify health policy frames within the documents, thereby going beyond the global-international health distinction and exploring more fine-grained framing categories. This also implied the elaboration of the two-layered typology (see below). Third, we systematically and comparatively applied the framework to the documents using NVivo. Codes were linked to each of the four frames and attributed to text parts that expressed arguments of the donor to engage in global health. For example, within NVivo the code ‘protect own population’ was linked to the security frame, ‘successful companies abroad’ to the investment frame, ‘health as a human right’ to the social justice frame, and ‘improve lives of the poorest people’ to the charity frame. This analysis was done by Lies Steurs and the research findings were discussed extensively with the co-authors, after which the main structure of the findings was decided. Fourth, where possible the findings were confronted with existing analyses of the health strategies (to the best of our knowledge only for the European Commission, the United Kingdom, and Germany some secondary literature was available^20,25,26,31^).

### A Typology of Global Health Frames

Global health is a complex policy area which is understood differently by academic scholars and policy makers. For this paper, we will not stick to a strict definition: in line with our constructivist ontology the assumption behind the study is indeed that actors engage in different definitions of the concept and that we should gain a better understanding of how European donors interpret global health. The first level of our typology relates how the external dimension of health policy is constructed. Here, the literature typically distinguishes between ‘international health’ and ‘global health.’^32,33^ The term ‘*international health*’ is associated mainly with assisting developing countries in fighting infectious and neglected tropical diseases, whereas ‘*global health*’ is understood as a broader concept focusing on the health impacts of deepened globalization for all countries (also industrialized countries)^ [3] ^. Institutionally, the former involves mainly development actors whereas the latter involves a larger range of actors. Before the global health revolution, international health policy was mainly dealt with by the ministries of development cooperation, growing out of former colonial relations. However, the increasing awareness of Western states’ own interests in global health has ‘lifted’ the subject onto the agenda of ministries of health and foreign affairs. In a growing number of countries, a ‘whole-of-government approach’ is used to address a broad range of global health themes.

More specifically there are different perspectives on the main problems (and solutions) involving ‘global health,’ in particular when it comes to health policy in the context of developing countries. In this article we refer to these as global health ‘frames,’ which constitute the second level of our typology. Previous research has identified several ‘frames,’ ‘perspectives’ or ‘metaphors’ of global health.^14,17,34-36^ Following the above-mentioned abductive approach, we distinguish four frames, namely *social justice, charity, investment* and *security.* Differences between these frames relate to four criteria: the purpose, main interest, commitment towards international health assistance (IHA), and the main focus (Table 2).

**Table 2:** Typology of Global Health Frames

**Frame**	**External Health**			
	**International Health**		**Global Health**	
	**Charity**	**Social Justice**	**Investment**	**Security**
Purpose	Fight absolute poverty	Reinforce health as a social value and a human right	Maximize economic development	Combat infectious diseases and contribute to social and political stability
Main beneficiary	Partner countries	Partner countries	Donor countries	Donor countries
Commitment towards IHA	Ad-hoc, unpredictable	Long-term	Long-term	Long-term
Main focus	Popular themes of victimhood and emergencies	Health systems and primary healthcare	Disease-specific	Disease-specific

Abbreviation: IHA, international health assistance.

The *charity* frame promotes health as a key element in the fight against poverty and prioritizes popular themes of victimhood such as mother and child mortality health and malnutrition.^35^ Lencucha links the charity frame also with the periodic engagement with events such as natural disasters or catastrophic events that pose an imminent threat to the health of people.^36^ Just like the social justice frame, it refers to the interests of the inhabitants of the countries receiving health assistance. However framed as charity, IHA is voluntary, temporary and reactive.^36^ The amount of IHA depends entirely on the benevolence or generosity of the contributor, which makes it less reliable than the social justice frame.

The *social justice* perspective aims to “reinforce health as a social value and human right, supporting the UN MDGs, advocating for access to medicines and primary healthcare, and calling for high income countries to invest in a broad range of global health initiatives.”^34^ It builds on cosmopolitan values that stress the importance of solidarity towards individuals at the global level, notwithstanding their nationality.^36^ According to this frame, the national government is not the sole responsible for realizing the right to health for its population, as countries ‘in a position to assist’ bear a complementary international obligation as well. The level of the health assistance should be based on the needs of the country and aims to fill the gap between what the national government can provide and what is needed to realize the right to health. The funding is for a considerable part focused on health systems and primary healthcare.

The *investment* frame considers health as a means of maximizing economic development.^35^ It is not only concerned with the economic effects of health on the population of countries receiving IHA, but also with the result of a growing global market in health goods and services.^34^ Compared to the two previous frames, the investment frame thus marks a shift the main beneficiaries being seen as the donors themselves instead of the partner countries: if IHA contributes to economic growth, the donors will benefit as well, as they will be able to export more products and services to the countries. This frame provides strong incentives for the continuation or even increase of IHA, but with a focus on the control of diseases that mostly affect the economically productive part of the population.

Similar to the investment frame, the *security frame* is also defined in a self-interested way, as the main beneficiaries are mainly seen to be the donor countries’ own population that needs to be protected. Global health funding can contribute in two ways to security: either by helping to contain infectious diseases in other parts of the world or by contributing to social and political stability (which might be at risk due to bad health conditions). The security frame motivates long-term action, following the logic that sustained support will ensure sustained national security.^36^ Nevertheless, security-based concerns lead to a main focus on infectious diseases. According to Rushton, health security could also be conceptualized in a less self-interested way, namely as a vital part of ‘human security,’ recognizing a broader range of threats and taking the individual/community as the primary referent object instead of the (western) state.^37^ However, the infectious disease-focused and state-centric version of health security is used more frequently.

The relation between the international/global health distinction on the one hand and different global health frames on the other hand has not been theorized in existing literature. Being two separate levels, we however expected some correlations between them. In ‘international health’ strategies, we expected the ‘charity’ and ‘social justice’ frames to be dominant, while ‘global health’ strategies would relate more to the ‘investment’ and ‘security’ frames. The reason for these expectations is two-fold. First, and from an interest perspective, ‘global health’ partly originated because of the increasing awareness of Western states’ own interests in external health policy, we could expect that the self-interested frames would be more dominant. Second, and from an institutional perspective, the actors involved in ‘global health’ strategies may be more interest-oriented than those involved in ‘international health’ strategies. Framing analysis pays specific attention to how actors and institutions frame issues in a particular way.^10,17^ We can expect that dominant frames will differ depending on which institution within the country takes the lead in formulating a global health policy. Since ‘international health’ policies are mainly developed by the development cooperation ministry, charity or social justice frames would be more dominant. On the other hand, ‘global health’ strategies are developed following a ‘whole-of-government approach,’ involving several ministries and departments. Here, the dominance of one or another frame may be linked to who had the biggest voice in the debate. However, the exact relationship between the two layers remains unclear; for instance there might be security concerns involved in ‘traditional’ ‘international health’ assistance whereas ‘global health’ strategies may also include social justice concerns. Ultimately, the two-layered typology needs to be examined more systematically as will be done in the next section.

## Results and Discussion

### The Notion of ‘Global Health’ Within the European Union

With regards to how the external dimension of health is perceived – the distinction between ‘international health’ and ‘global health’ – we find that there are two groups among the selected donors and that this corresponds to the institutional actors involved in the formulation of the strategy.

First, the European Commission and three Member States, namely the United Kingdom, Germany, and France, have developed a ‘global health’ strategy over the past years. These policy documents focus on the health impacts of globalization, the shared risks and threats and the need for a truly global action. The global health policy documents were developed by several Ministries or Directorate-Generals and were therefore presented as whole-of-government strategies.

Within the United Kingdom the Department of Health led an inter-ministerial working group for Global Health, which coordinated the development of the 2008 strategy and would oversee its implementation.^38^ The group included representatives of a wide range of departments^ [4] ^, with the Department of Health, the Ministry of Defense, Department for International Development (DFID) and the Foreign and Commonwealth Office being the most important. For the development of the German strategy several ministries were involved as well claiming that “the federal ministries involved already regularly share their information and experience on current and planned activities in the field of global health. When needed, this instrument will be expanded.”^39^ However, it is unclear from the Strategy which ministries are actually involved. Bozorgmehr and colleagues also criticized the lack of clarity on how the inter-ministerial collaboration would be effectively arranged.^31^ In the case of France, we could literally see the evolution from ‘international health’ towards ‘global health.’ In 2012, the Directorate-General of Global Affairs, Development and Partnerships of the French Ministry of Foreign and European Affairs developed an ‘international health’ strategy. While stating that “the important pace of globalization has increased the cross-cutting nature of health threats and demonstrated the shared benefits of universal access to quality care,”^40^ the strategy focuses only on the development aspects of health. In 2017 however, a ‘global health’ strategy was developed, led by the same Directorate General, but in close collaboration with other ministries and agencies^ [5] ^. The 2010 Communication of the European Commission was launched by three directorates-general, namely DG Devco, DG Sanco and DG Research. These three DGs are also taking the lead in the further development of global health action of the EU as rotating chairs of an inter-service group on global health.^20^

However, even within the documents of those countries that do refer to ‘global health,’ there is not a clear definition of what the concept exactly entails. Only within the global health strategy of the United Kingdom a definition is mentioned. The European Commission on the other hand, claims that “no single definition of the concept exists”^6^ and the German and French strategies do not elaborate on the definition of the concept.

The second group consists of Belgium and Denmark, who do not have ‘global health’ strategies. They do have policy notes on the health sector however, developed respectively by DG Development of the Federal Public Service for Foreign Affairs, Foreign Trade and Development Cooperation and DANIDA (the Danish International Development Agency) of the Ministry of Foreign Affairs of Denmark. Given this institutional set-up and the exclusive focus on development cooperation, these strategies can be considered to be international health strategies.

While the United Kingdom and Germany developed a global health strategy in respectively 2008 and 2013 we also analyzed the ‘DFID health position paper’ (2013) and the Sector Strategy on German Development Policy in the Health Sector (2013), which can be considered to be ‘international health’ strategies. Interestingly, the ‘international health’ strategy from DFID was developed after the ‘global health’ strategy. Nevertheless, the DFID position paper did not replace the global health strategy, as “it stops short of being a full health strategy and so does not contain new policy or a full reflection of the whole of the UK government’s health investments in developing countries.”^41^

While European member states such as Belgium and Denmark do not have a ‘global health’ strategy, this does not necessarily imply that their external health policies and practices remain restricted to those of the development department. Therefore, the development of a ‘global health’ strategy could make all actors involved more aware of each other’s policies and responsibilities and could therefore contribute to more coherence and visibility of the country’s engagement in global health.

As suggested in the previous section, we expect that the fact that some countries have a ‘global health’ strategy, while others only have an ‘international health’ strategy, also impacts on the framing of the policy documents, as will be discussed in the next part.

### Differences in Framing

When looking at the frames within the policy documents, we identified 4 main findings which will be explained in the following sections.

#### 1. Absence of Charity Frame

First, the charity frame was almost non-existent in the policy documents. There are occasionally some small references in the documents that could be linked to this frame, for example “one way for us to build a better, fairer world,”^42^ or “an expression of solidarity with the countries.”^40^ In general however, the charity frame is relatively absent. This might perhaps not be a surprise as the international development discourse of official donors has moved away from charity considerations, which may be expected more from some non-governmental (including religious and philanthropic) organizations involved in development.

Interestingly, however, the difference between the charity frame and the social justice frame – which is much more prominent – can sometimes be quite subtle. When referring to human rights, this implies a responsibility and obligation for countries in a position to assist supporting countries to fulfill these rights. However, when referring to values such as solidarity and equality, there is a risk that providing assistance becomes more optional and voluntary. The latter leans more towards the charity frame.

#### 2. Dominance of Social Justice Frame in “International Health” Documents

The social justice frame is present in the policy documents of all donors. However, it is most explicit in the international health strategies of all donors, as well as in the global health strategy of the European Commission.

Firstly, these documents often refer to *health as a human right*. This can clearly be seen in the policy document of Belgium, which is entitled ‘right to health’ and focuses entirely on how to achieve this** “**inalienable right.”^43^ The German sector strategy of 2009 also had a human rights approach, explicitly claiming that “German development policy in the health sector pursues a human rights-based approach.”^44^ Within the DFID strategy it was mentioned that “first and foremost, better health is an end in itself and a basic human right.”^41^ For Denmark “the rights issue is key,”^45^ especially when it comes to sexual and reproductive health and rights and in the France international health document human rights are mentioned as a central value. Lastly, the EU’s focus on strengthening of health systems should lead to “basic equitable and quality healthcare for all without discrimination on any grounds as defined by Art. 21 of the Charter of Fundamental Rights.”^6^

Secondly, some of these strategies make reference to certain *values and principles* in addition to rights. For example, the European Commission states that “the EU should apply the common values and principles of solidarity towards equitable and universal coverage of quality health services in all external and internal policies and actions.”^6^ The international health strategy of France mentions solidarity, human rights and aid effectiveness as the central values of their strategy, which are all three clearly linked with the social justice frame.^40^ However, the strategy does not elaborate much on the specific understanding of these values.

While the social justice frame is dominant in the ‘international health’ strategies and the Commission communication, there are occasional references to the investment and security frames as well in some of the documents. Nevertheless, this is less prominent than is the case in the ‘global health’ strategies of France, the United Kingdom, and Germany. For example, the communication of the European Commission often refers to the EU’s leading role in international trade, stating that this gives the EU “strong legitimacy to act on global health,”^6^ but not explicitly referring to the global health-related trade interests for the EU. The DFID strategy also links the contribution of better health to “higher productivity and hence economic growth,”^41^ but does not link this with UK interests. The international health strategy of France refers to the ‘collective health security,’ without explicitly referring to the protection of its own citizens.^40^

As we will discuss in the next part, the social justice frame also appears to a certain extent in the ‘global health’ policy notes of the United Kingdom, Germany, and France, but the security and/or investment frames are more dominant in these documents. This seems to correlate with differences in institutional set-up, as could also be expected from a framing analysis perspective. The ‘international health’ strategies were developed within the development department only. An exception to our initial expectation would be that social justice features prominently in the Commission’s ‘global health’ strategy. This could be explained by the fact that, although the communication of the European Commission was launched by three DGs, there was only one commissioner taking the lead, which happened to be the development commissioner.

#### 3. Additional Security and Investment Frames in Global Health Documents

Within the German, British and French global health policy documents, there is a more ‘fuzzy’ combination of frames. The following quotes from the introductions of the strategies illustrate the explicit reference to different frames.

*“Global Health interacts with all core functions of foreign policy: achieving national and global security, creating economic wealth, supporting development in low-income countries and promoting human dignity through the promotion of human rights and the delivery of humanitarian assistance.”* ^46^

*“Health is not only a fundamental human right and one of the most valuable possessions any individual can have, it is also an essential prerequisite for social, economic and political development and stability.”* ^39^

*“The diversity of the actors involved makes it possible to take into account the diversity of health approaches: development and solidarity, economic diplomacy, scientific diplomacy, attractiveness, security, bilateral cooperation and multilateral negotiations, academic and training exchanges, research, etc.”* ^47^

Again, the institutional factor, namely which ministry is taking the lead within the countries, sheds light on these findings. The additional focus on security and investment in the global health policy documents of Germany, the United Kingdom, and France can indeed partly be explained by the fact that several ministries were on the table. Ministries of health and/or foreign affairs would have focused mostly on the security aspects, while Ministries of Foreign policy and trade would also be stressing the investment arguments. The fact that the ‘international health’ strategies of these countries do not focus much on investment and security confirms this analysis, as these strategies were only developed by the development departments. The DFID health policy position paper of 2012 was even developed after the ‘Health is Global Strategy’ and mainly focused on health as a human right without referring to the security interests of or investment opportunities for the United Kingdom.

While the German, British, and French strategy are all combining the three frames, there are nevertheless some differences with regards to how the different frames are balanced against each other and to what extent self-interest is emphasized.

*The French strategy is balancing the three frames equally*. There are several references to human rights and values such as solidarity and equality, which link to the social justice frame. The security frame is present as well, given that “strengthening international health security”^47^ is one of the priorities. As was the case within the international health strategy of 2012, the focus is however more to “protect the world’s population”^47^ instead of protecting mainly the French population. On top of that, health security is explicitly linked to the strengthening of health systems. When it comes to the investment frame, there is a focus on how the French expertise related to health – referred to as “the brand French healthcare”^47^– can be promoted to improve global health.

*Within the German strategy the self-interest frames are slightly taking the upper hand.* There are several references to the human right to health and values such as universality, solidarity, access to high quality healthcare and equal treatment, which links to the social justice frame. Nevertheless, there is also a clear focus on the security and investment frames, often linked to the self-interest for Germany. The security arguments are mainly focused on the protection against cross-border health threats claiming that one of the goals of the strategy is to “ensure the sustainable protection and improvement of the health of the German Population.”^39^ The investment frame is also clearly present, as one of the focus areas is “health research and the health industry,”^39^ where it is stated that “German health research and the healthcare industry […] can make an essential contribution to improving the global health situation.”^39^ Within existing literature, this focus on domestic economic interests was criticized, as the strategy does not consider global debates on intellectual property rights and access to medicines.^26,31^

Even more so than the German strategy, the *British global health documents are very much focused on self-interest.* The 2008 ‘Health is Global’ strategy fits the social justice frame as it makes a reference to human rights. However, as Labonté and Gagnon pointed out, the most prevalent objective of this strategy is to benefit the United Kingdom.^25^ One of the criteria used to determine the areas covered in the 2008 ‘Health is Global’ strategy was “whether the United Kingdom stands to benefit directly from engaging in the issue, for example, where there are clear links to the health of the UK population.”^42^ Accordingly, there is a clearly dominant focus on security and investment within the strategy. As mentioned in the foreword written by Gordon Brown, “global health is a question not just of morality but of security as well. […] the first duty of any government must be to ensure the safety of its people, but this can no longer be achieved in isolation.”^42^ Interestingly, the security arguments not only focus on protecting the population against infectious diseases, but there is also much attention for possible threats of political instability elsewhere for the United Kingdom.

*“Poor health is more than a threat to any one country’s economic and political viability – it is a threat to the economic and political interests of all countries. Working for better global health is integral to the UK’s modern foreign policy.”* ^42^

This quote illustrates that the focus is not only on political stability but also on economic stability. This connects with the investment frame, which can be derived from two main arguments made in the document. Firstly, there are many references to the link between a healthy population and economic stability, productivity and growth. Secondly, the specific economic benefits for the UK health industry are stressed with one of the objectives being “the enhancement of the United Kingdom as a market leader in well-being, health services and medical products.”^42^ The dominance of the security and investment frames was even more apparent in the 2011 outcomes framework, which aimed to “reassure the UK’s security and prosperity at home, and UK citizens’ interests overseas.”^46^ Furthermore, while there was still a brief reference to human rights in the 2008 document, there was none in the framework.

Our findings confirm our expectation that global health strategies would focus more on the economic and security frames. On the one hand, this could have the positive effect of lifting the topic of global health higher on the policy agenda and increasing the visibility of a country’s engagement in global health. As mentioned before, despite the moral obligations to fulfill and respect human rights, there is still a great level of ‘optionality’ and ‘voluntariness’ involved with the social justice frame. The security and investment frames that focus more on the self-interest for Western countries could potentially make it more easier to legitimize the need to invest in Global health. Perhaps not accidently, Germany, the United Kingdom, and France are among the most prominent funders of global health initiatives. However, countries engaging in a global health approach should be careful that interest-based motives (security and investment) are in balance with social justice considerations. Otherwise there is the possibility of foreclosing certain areas of action that are of less interest for countries providing assistance.

## Conclusion

Global health has appeared highly on the international agenda over the past two decades. Also the EU and most of its member states have been active in this area. The European Commission has been and remains an important actor in global health, while at the same time common strategies for global health have been developed at the EU level. Nevertheless, member states remain important bilateral players in this field as well. Against this backdrop, this article aimed to analyze to what extent a common ‘EU’ vision in global health might have emerged? In order to answer this question, we engaged in a framing analysis of relevant policy documents of the European Commission, the United Kingdom, France, Denmark, Belgium, and Germany.

It became clear that the concept of ‘global’ health has not yet gained ground within the policy documents of all member states. The European Commission and three Member States, namely the United Kingdom, Germany, and France, developed a ‘global health’ strategy over the past years. These were elaborated through a whole-of-government process and have a comprehensive approach. In contrast, the relevant policy documents of Belgium and Denmark were elaborated within the development departments and focused only on developing countries, implying that these can be considered to be ‘international health’ strategies. Taking this into account, the framing analysis has shown a mixed picture regarding the existence of an ‘EU’ vision. The ’international health’ strategies and the communication from the European Commission clearly hold a social justice frame, stressing values and human rights. While the social justice frame is also apparent, at least to some extent, in the ‘global health’ strategies of the United Kingdom, Germany, and France, the security and/or investment frames are more dominant in these documents. There are nevertheless differences in the extent of how much self-interest is stressed within the strategies.

Based on these findings, we can conclude that the existence of an ‘EU’ vision on global health is questionable. This is coherent with the analysis of Battams and Van Schaik that global health created potential tensions for both the coherence between EU member states and EU institutions, as well as the coherence between health policy experts and specialists in other areas such as development or foreign policy.^48^

The absence of a single EU frame for global frame may not be problematic from a normative perspective – even if it goes against ambitions formulated by the European Commission. However, if the EU wants to be a credible and effective actor in global health, there is a need for European policy-makers to engage more in deliberations on what exactly their global health policies imply. In this context, member states that hold to a traditional ‘international health’ approach could consider the modernization towards a global health approach, in line with advances in global health thinking at the level of the EU, the WHO, and other international institutions, whereas countries engaging in a global health approach should be careful that interest-based motives (security and investment) do not out-balance social justice considerations. Such deliberations would at least foster further debate on how global health could and should be pursued by important donors such as the EU and its member states and how synergies between different actors and approaches could be pursued. Given the diversity in approaches, a ‘division of labor’ that acknowledges existing differences between the EU and its member states may be more feasible than a ‘common’ EU policy.

Building on these insights, whilst addressing some limitations of this article, further research is needed on several aspects. First, more explanatory research could be done on the observed differences between Member states. Why are the United Kingdom, Germany, and France the only member states with a global health strategy? And how can we explain the differences in framing? We highlighted differences in institutional set-up as one explanatory factor, but other factors related to domestic politics should also be considered. Second, the implementation and operationalization of the ‘global’ health/’international’ health strategies should be examined. While we reflected on the potential foreclosing of certain focus areas due to certain framings, more empirical research should be conducted to analyze whether and how different framings by member states indeed translate into different political practice. Several case studies could be examined in this regard, for example, the EU and member states’ approaches to the recent Ebola outbreak in West-Africa. Third, future research could look into coordination (eg, a ‘division of labor’) on global health between EU member states. A critical investigation of Brussels-based coordination mechanisms such as the EU Member states Experts Group on Global Health, Population and Development might be helpful in this regard, in addition to field work on European coordination and division of labor within developing countries. Fourth and finally, the two-layered typology could be further theorized, benefiting from extended research on global health policies of other member states.

## Acknowledgements

Lies Steurs has received a VLIR-VLADOC scholarship (2014-2018) from the Flemish Government for her doctoral research.

This manuscript builds on the working paper “International assistance from Europe for global health: searching for a common paradigm” (2012) of our colleagues Gorik Ooms, Rachel Hammonds, and Wim Van Damme from the Institute of Tropical Medicine, Antwerp, Belgium. We are grateful for their insight and advice, on which we could build our conceptualization and analysis.

## Ethical issues

Not applicable.

## Competing interests

Authors declare that they have no competing interests.

## Authors’ contributions

All authors conceived the study; LS wrote a first draft. All authors contributed with inputs and review. All authors agree on publication.

## Authors’ affiliations

^1^Centre for EU Studies, Ghent University, Gent, Belgium. ^2^Institute of Tropical Medicine, Antwerp, Belgium. ^3^Centre for Global Health Research, Maastricht University, Maastricht, The Netherlands. ^4^Clingendael, Netherlands Institute of International Relations, The Hague, The Netherlands.

## Endnotes


[1] For example, GAVI, the Vaccine Alliance in 2000, the Global Fund to Fight AIDS, Tuberculosis and Malaria in 2002 and the US President’s Emergency Plan For AIDS Relief (PEPFAR) in 2003.

[2] For Denmark, we also analyzed the specific strategies for HIV/AIDS and sexual and reproductive health and rights, as the general health and development policy note mentions that the Danish development assistance in health is underpinned by these two strategies.

[3] For reasons of simplicity, the term global health is used in this article as an overarching term for both ‘international’ and ‘global’ health. When referring to the semantic difference between both terms, the terms ‘international’ and ‘global’ health will be put between quotation marks.

[4] The working group includes Ministers from the Department for Business, Enterprise & Regulatory Reform, Department for Children, Schools and Families, Department for Environment, Food and Rural Affairs, Ministry of Defense, Department of Health, Department for Innovation, Universities & Skills, DFID, Foreign and CommonwealthOffice, Home Office, HM Treasury, and the Northern Ireland Government.

[5] The Ministry of Social Affairs and Health and its agencies, the Ministry of Higher Education and Research, the Ministry of Economy and Finance, the Ministry of Agriculture, Food and Forestry, L’Agence Française de Développement (AFD) and Expertise France.


## Key messages

**Implications for policy makers**

- Global health is a not well-defined policy concept. Consequently, it has been framed in different ways by the European Union (EU) and its member states.
- The European Commission and its member states have different policies to further global health objectives. While some of them take a more comprehensive approach combining domestic and foreign policy objectives, others maintain an international health approach to be pursued via development cooperation.
- The existence of a common ‘European’ vision on global health is questionable. Before outlining a so-called EU vision on global health, European policy-makers should engage in deliberations on what exactly their global health policies imply.
- European member states that still hold to a traditional ‘international health’ approach should consider the modernization towards a ‘global health’ paradigm, in line with advances in global health thinking at the level of the EU, the World Health Organization (WHO) and other international institutions.
- Countries engaging in a global health approach should be careful that interest-based motives (security and investment) are in balance with social justice considerations.
- While the EU is increasingly trying to coordinate the European action in global health it is not clear yet where these coordination efforts are leading to. Given the diversity in approaches, a ‘division of labor’ that acknowledges existing differences between the EU and its member states may be more feasible than a ‘common’ EU policy.

**Implications for the public**

Global health policy development by the European Union (EU) and its member states has been pursued in diplomatic, administrative and professional policy-maker’s circles. While the general public may be actively involved in policy dialogue on health policies at the national level, such as in the field of health insurance and budgetary choices, this is much less the case when it comes to global health objectives. Nonetheless, it has been increasingly recognized that global health issues also affect the population of European countries (for instance, but not exclusively, trough risks related to epidemics such as Ebola). The EU’s democratic legitimacy problem is also seen in the global health domain. If the EU and its member states aim to further advance an ‘EU role in Global Health’ it is important to facilitate dialogue on health priorities, strategies and policies at the national and EU level. Deliberation should include civil society, professional networks and citizens in general, within the EU as well as abroad, as health challenges are transnational and require close cooperation to overcome them.
